# Supplementary material for: Comprehensive patient-level classification and quantification of driver events in TCGA PanCanAtlas cohorts
Source: PLoS Genet. 2022 Jan 14;18(1):e1009996. doi: 10.1371/journal.pgen.1009996 (PMC8759692; doi:10.1371/journal.pgen.1009996)
Supplement: S4 Files — (ZIP) [file pgen.1009996.s004.zip › Aneuploidy/COHORTS GISTIC2/patient distributions/2021_11_23_15_0_PCPG_FEMALE.pdf]

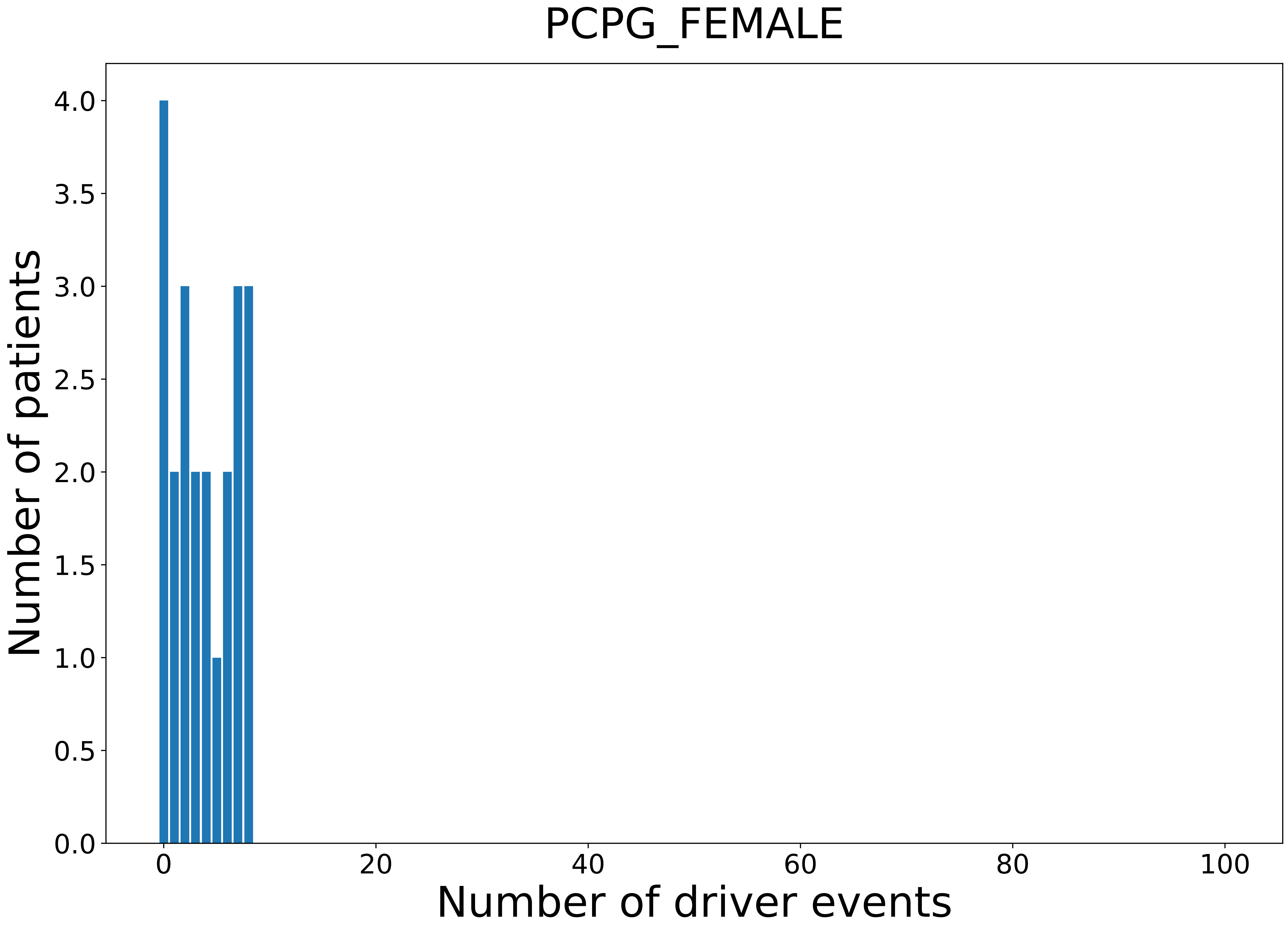
PCPG_FEMALE
Number of patients
Number of driver events
4.0
3.5
3.0
2.5
2.0
1.5
1.0
0.5
0.0
0
20
40
60
80
100
